# Retinal Thickness Analysis Using Optical Coherence Tomography: Diagnostic and Monitoring Applications in Retinal Diseases

**DOI:** 10.3390/diagnostics15070833

**Published:** 2025-03-25

**Authors:** Seong Joon Ahn

**Affiliations:** Department of Ophthalmology, Hanyang University Hospital, Hanyang University College of Medicine, Seoul 04763, Republic of Korea; ahnsj81@gmail.com; Tel.: +82-2-2290-8574

**Keywords:** diagnosis, monitoring, optical coherence tomography, retinal disease, retinal thickness

## Abstract

Retinal thickness analysis using optical coherence tomography (OCT) has become an indispensable tool in retinal disease management, providing high-resolution quantitative data for diagnosis, monitoring, and treatment planning. This analysis has been found to be particularly useful for both diagnostic and monitoring purposes across a wide range of retinal diseases, enabling precise disease characterization and treatment evaluation. This paper explores its applications across major retinal conditions, including age-related macular degeneration, diabetic retinopathy, retinal vein occlusion, and inherited retinal diseases. Emerging roles in other diseases such as neurodegenerative diseases and retinal drug toxicity are also highlighted. Despite challenges such as variability in measurements, segmentation errors, and interpretation difficulties, advancements in artificial intelligence and machine learning have significantly improved accuracy and efficiency. The integration of retinal thickness analysis with telemedicine platforms and standardized protocols further underscores its potential in delivering personalized care and enabling the early detection of ocular and systemic diseases. Retinal thickness analysis continues to play a pivotal and growing role in both clinical practice and research, bridging the gap between ophthalmology and broader medical fields.

## 1. Introduction

Retinal diseases are a leading cause of vision impairment and blindness worldwide, posing a substantial burden not only on individuals but also on healthcare systems and society at large [1,2]. Common conditions such as age-related macular degeneration (AMD), diabetic retinopathy (DR), and retinal vein occlusion significantly impact patients’ quality of life and contribute to substantial socioeconomic costs due to vision loss and associated disabilities [3,4]. Early diagnosis and timely intervention are crucial to mitigate these effects and preserve vision, emphasizing the need for reliable tools to detect and monitor retinal pathologies.

The advent of OCT has revolutionized the field of ophthalmology, offering unparalleled insights into retinal structure and thickness [5,6]. This non-invasive imaging modality provides high-resolution cross-sectional views of the retina, enabling the precise measurement of retinal thickness [7]. Retinal thickness analysis, in particular, has emerged as a pivotal tool for both diagnosing retinal diseases and tracking their progression over time [8]. This technique has enhanced our ability to identify subtle pathological changes, assess treatment efficacy, and predict visual outcomes [9].

This review aims to provide a comprehensive overview of the role of retinal thickness analysis using OCT in the diagnosis and monitoring of retinal diseases. We will explore the principles underlying retinal thickness measurement, the utility of retinal thickness maps, and the significance of sequential analysis in longitudinal monitoring. Furthermore, we will discuss the diagnostic and monitoring applications across a spectrum of retinal diseases, the challenges and limitations of the technique, and recent advances in imaging and analysis technologies. By synthesizing current knowledge, this review will highlight the critical role of retinal thickness analysis in advancing personalized treatment strategies and improving clinical outcomes in retinal disease management.

## 2. Retinal Thickness Analysis: An Overview

Retinal thickness analysis has become an essential component of modern ophthalmology, offering critical insights into the structural integrity of the retina. This section provides an overview of its principles, the generation of retinal thickness maps, and the importance of sequential analysis in tracking disease progression and treatment efficacy.

### 2.1. OCT Systems

Table 1 provides a concise comparison of key OCT systems used for retinal imaging, along with commercial examples. This summary highlights the primary technological differences, axial resolution, advantages, and limitations of each system, serving as a quick reference for understanding their clinical applications.

### 2.2. Definition and Principles of Retinal Thickness Analysis

Retinal thickness refers to the distance between the inner and outer boundaries of the retina, commonly measured from the internal limiting membrane (ILM) to the retinal pigment epithelium (RPE). Typically measured using OCT, retinal thickness serves as a key biomarker for both normal and diseased states of the retina, with changes in thickness often correlating with disease severity or treatment response [9].

Previous studies have employed a variety of parameters for analyzing retinal thickness and related measurements (Table 2), including mean central subfield thickness (CST) [10,11,12,13], macular volume [13,14], and segmented layer thicknesses [15,16]. Advancements in OCT technology, particularly the introduction of automated retinal layer segmentation, have enabled the precise measurement of individual retinal layers. These advancements enhance the ability to pinpoint structural changes specific to particular layers, improving diagnostic and monitoring capabilities.

### 2.3. Retinal Thickness Maps

**Figure 1:**
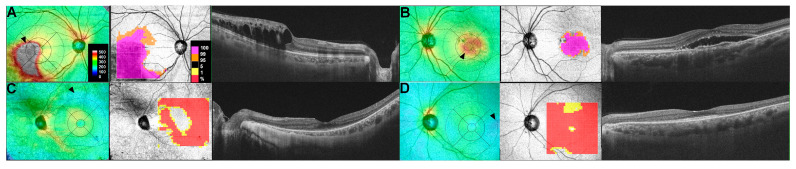
Composite images illustrating retinal thickness maps (**left**), retinal thickness deviation maps (**middle**), and corresponding OCT B-scan images (**right**) for various retinal pathologies: macular edema secondary to branch retinal vein occlusion (**A**), exudative AMD (**B**), retinitis pigmentosa (**C**), and hydroxychloroquine retinopathy (**D**). In these images, arrowheads highlight areas of altered retinal thickness, demonstrating how deviations from normal topography and thickness correlate with specific disease processes and thereby aid in diagnosis and monitoring.

Retinal thickness map bs are generated by analyzing OCT data across a grid pattern that encompasses the macula or wider areas. These maps provide a topographic representation of retinal thickness, offering a visual depiction of structural variations or thickness changes (Figure 1). In most OCT image viewer software, thickness maps use a color scale to represent variations, where warmer colors (e.g., red, orange) indicate areas of increased thickness and cooler colors (e.g., blue, green) denote thinner regions.

The OCT system can also segment the retina into its constituent layers (e.g., ILM, GCL, IPL, RPE). Layer-specific maps can highlight abnormalities within particular layers, aiding in the detection of disease-specific patterns such as subretinal fluid in AMD or the thinning of the outer nuclear layer in inherited retinal diseases [17,18].

In addition to absolute thickness maps, deviation maps compare an individual’s retinal thickness to a normative database [19,20]. These maps visually represent areas where retinal thickness deviates significantly from the normal range (Figure 1), helping identify early or subtle pathological changes. Areas of significant deviation are typically highlighted in contrasting colors, making it easier to detect abnormalities such as macular edema or atrophic thinning.

These thickness maps are widely used to diagnose retinal diseases and evaluate treatment efficacy, offering an intuitive method to assess thickness changes with spatial information. However, thickness maps need to be interpreted in conjunction with their corresponding structural OCT images. Although thickness maps provide a valuable topographic overview of retinal thickness variations, structural OCT offers the detailed anatomical context necessary to identify potential artifacts or segmentation errors, which may cause errors in thickness maps. This integrated assessment has enhanced diagnostic accuracy by ensuring that any abnormal thickness measurements are precisely correlated with the underlying retinal architecture, thereby mitigating the risk of misinterpretation.

### 2.4. Sequential Retinal Thickness Analysis

**Figure 2:**
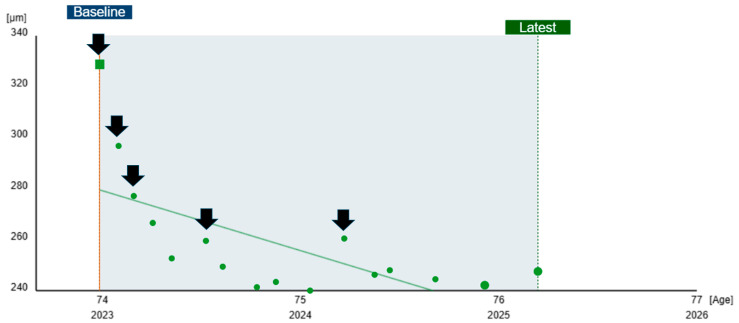
Sequential retinal thickness plot in an eye with exudative AMD treated with three loading anti-VEGF doses followed by as-needed injections, showing a marked post-loading reduction and progressively longer inactive periods over time, as indicated by the intervals between arrows (representing injections).

Sequential retinal thickness analysis involves tracking changes in retinal thickness over time, providing critical information about disease progression and treatment response [9]. Sequential analysis requires serial OCT scans, which capture changes in retinal thickness, enabling clinicians to detect subtle trends, such as progressive thinning in inherited retinal diseases or improvement in macular edema after anti-VEGF therapy (Figure 2) [9,21]. Additionally, some OCT systems offer automated trend analysis that visualizes thickness changes across multiple visits, facilitating longitudinal monitoring.

Sequential thickness analysis is crucial for its ability to sensitively track disease progression or recurrence, evaluate treatment efficacy, and predict structural or functional outcomes [22,23,24]. For instance, progressive retinal thinning may signal advancements in inherited or degenerative retinal diseases, while retinal thickening in eyes with a history of macular edema could indicate a recurrence of pathology [22,23]. Additionally, reductions in retinal thickness following treatments, such as anti-VEGF injections, serve as an indicator of therapeutic response [25,26]. Changes in retinal thickness can also correlate with functional outcomes, such as visual acuity, offering insights into functional recovery and aiding clinical decision-making [21].

Combining retinal thickness maps with sequential analysis provides clinicians with a more comprehensive understanding of retinal diseases by integrating spatial patterns and temporal trends, enabling the development of more effective management strategies.

### 2.5. Inter-Device Variability and Standardization

Inter-device variability is a critical issue in retinal thickness analysis, because differences in OCT devices, scan protocols, segmentation algorithms, and normative databases can lead to significant discrepancies in measurement. These variations may influence clinical decision making by affecting diagnostic thresholds, treatment monitoring, and the interpretation of disease progression, particularly when comparing serial measurements obtained from different devices.

Several standardization approaches have been proposed to mitigate these challenges [27,28]. These include cross-device calibration techniques that establish conversion factors or calibration protocols to adjust measurements between devices, thereby improving consistency and enabling reliable comparisons across different OCT systems; deep learning-based harmonization methods that use advanced artificial intelligence techniques to learn device-specific biases and harmonize data, reducing variability and improving the accuracy of retinal thickness assessments; and consensus guidelines that involve developing and adopting standardized protocols for OCT acquisition and analysis, including uniform segmentation algorithms and normative databases.

Implementing these strategies is essential to enhance measurement reliability, support accurate clinical decision making, and ultimately improve patient outcomes in the management of retinal diseases.

## 3. Applications of Retinal Thickness Analysis in Various Diseases

Retinal thickness analysis using OCT has become an indispensable tool for both diagnosing and monitoring various retinal diseases. Its ability to provide high-resolution, quantitative data enables early detection, disease characterization, and treatment response evaluation. This section elaborates on its applications across major retinal pathologies, emphasizing its roles in diagnosis and disease monitoring, as summarized in Table 3.

### 3.1. AMD

Retinal thickness analysis plays a pivotal role in managing AMD by distinguishing disease subtypes and tracking therapeutic responses [29].

In AMD, OCT identifies hallmark features such as subretinal fluid, pigment epithelial detachment (PED), and thickening of the retina in wet AMD [30]. Retinal thickness maps also differentiate wet AMD from the dry subtype by highlighting fluid or hemorrhage accumulation, represented by a thickness increase caused by neovascular activity [9]. Such features are instrumental in guiding initial treatment decisions.

Serial retinal thickness measurements are used to evaluate the efficacy of anti-VEGF therapy, with reductions in fluid and central retinal thickness indicating a positive response [9]. The longitudinal monitoring of the outer retinal layers, particularly the RPE and photoreceptors, helps predict visual outcomes and assess progression to geographic atrophy in advanced cases [31].

### 3.2. DR and Diabetic Macular Edema (DME)

Retinal thickness analysis is essential in the detection and management of DR and DME, where edema is a key complication.

OCT detects increased CST, as well as qualitative features such as intraretinal and subretinal fluid, which are hallmarks of macular edema [32,33]. These features enable the identification of CSME even in asymptomatic patients, facilitating timely intervention.

Serial OCT imaging assesses treatment response to intravitreal anti-VEGF or steroid injections [34,35]. Persistent thickening or recurrent fluid suggests a suboptimal response, guiding re-treatment decisions. Additionally, retinal thickness and deviation maps allow clinicians to identify areas of residual or progressive edema.

### 3.3. Retinal Vein Occlusion (RVO)

Macular edema, a frequent complication of RVO, is effectively managed using retinal thickness analysis.

OCT identifies cystoid spaces, subretinal fluid, and diffuse macular thickening, providing detailed features associated with macular edema and the severity of branch or central RVO [36,37,38]. Furthermore, retinal thickness maps may be utilized for the early detection and prediction of the recurrence of macular edema and the need for re-treatment [39]. These findings help guide initial treatment, as well as confirming the diagnosis.

Treatment efficacy, particularly with anti-VEGF or corticosteroid therapy, is monitored through reductions in retinal thickness and the resolution of fluid [39]. Sequential imaging allows clinicians to identify recurrence or its trend, ensuring optimal disease management.

### 3.4. Inherited Retinal Diseases

In progressive inherited retinal disorders such as retinitis pigmentosa and Stargardt disease, retinal thickness analysis provides valuable structural and functional insights [40,41].

The thinning of specific retinal layers—such as the outer nuclear layer and photoreceptors—or macular volume serves as a biomarker for progression and its rate [41,42]. Such changes detected via OCT also correlate with phenotypic disease entities and underlying genetic mutations [42].

Longitudinal analysis reveals the trajectory of disease progression, supporting genetic counseling and patient selection for clinical trials targeting retinal regeneration or gene therapy [43].

### 3.5. Central Serous Chorioretinopathy (CSC)

CSC is characterized by serous retinal detachment and RPE changes, which are readily identified using OCT [44]. These features also help differentiate CSC from similar conditions, such as AMD. Sequential OCT imaging and temporal patterns of thickness changes may be useful for the detection or prediction of its recurrence and the assessment of spontaneous resolution or response to treatments such as photodynamic therapy [45].

### 3.6. Uveitis and Uveitic Macular Edema

Macular edema resulting from intraocular inflammation is another condition where retinal thickness analysis proves invaluable. OCT reveals increased total retinal thickness or RNFL thickness in active uveitis, regardless of etiology [46], including cases associated with HLA-B27 [47], highlighting retinal thickness as a reliable biomarker of uveitis activity.

Therefore, sequential OCT imaging may play a role in monitoring treatment responses to corticosteroids, immunomodulatory agents, or biologics, and in detecting early recurrence. A reduction in retinal, particularly peripapillary and perivascular, thickening, along with the resolution of cystoid spaces, serves as a reliable indicator of therapeutic efficacy and disease control, whereas persistent or recurrent thickening may signal a suboptimal response or persistent inflammation, prompting adjustments to the treatment regimen.

### 3.7. Myopic Maculopathy

High myopia is associated with structural retinal changes that predispose individuals to vision-threatening complications such as myopic neovascularization and macular atrophy. Retinal thickness is associated with refractive error and axial length, with regional variations [48], and OCT identifies characteristic thinning of the inner and outer retinal layers, as well as complications such as myopic traction maculopathy and macular holes. Progressive thinning, retinal detachment, or the development of complications can be detected or monitored through retinal thickness analysis, enabling timely surgical or pharmacological intervention.

### 3.8. Pediatric Retinal Diseases

With advances in handheld OCT, OCT imaging is increasingly applied to pediatric and neonatal populations, offering valuable insights into the structural and developmental changes in the retina. In preterm infants, handheld OCT provides a detailed visualization of abnormal retinal development, including changes in thickness and photoreceptor integrity [49]. Sequential imaging enables the monitoring of retinal maturation and the progression of retinopathy of prematurity (ROP), identifying complications such as preretinal neovascularization and retinal detachment [50].

Beyond ROP, retinal thickness analysis is also valuable in diverse pediatric retinal and optic nerve conditions. For example, optic pathway gliomas are associated with neurofibromatosis type 1 (NF1), where retinal nerve fiber layer thinning can serve as an indicator of disease progression or optic nerve dysfunction [51]. In hereditary retinal diseases, such as Leber congenital amaurosis and retinitis pigmentosa, OCT thickness analysis may facilitate the monitoring of outer retinal abnormalities, such as disruption or thinning of the ellipsoid zone, which can guide genetic testing and counseling [52,53,54]. These applications highlight the growing role of OCT thickness analysis in understanding and managing a wide spectrum of pediatric retinal conditions.

### 3.9. Retinal Drug Toxicity

Retinal thickness analysis is increasingly utilized to detect and monitor retinal toxicity caused by systemic or ocular drugs. Certain medications are known to induce structural changes in the retina, which can compromise visual function if not detected early.

Retinal thickness analysis using OCT provides high-resolution imaging to detect subtle, drug-induced changes in the retina [20]. For instance, hydroxychloroquine toxicity often manifests as localized thinning of the posterior pole, particularly in the parafoveal and pericentral areas, with disruption or loss of the photoreceptor layer with or without RPE changes [20,55]. Pentosan polysulfate maculopathy is also characterized by outer retinal degeneration, including paracentral thinning of the retina, particularly in the photoreceptor and RPE layers [56]. Early detection through OCT and thickness analysis enables intervention before irreversible vision loss occurs. However, tamoxifen-related toxicity may result in crystalline deposits and retinal thickening [57].

Thus, serial OCT imaging is invaluable in assessing the retinal changes associated with prolonged medication use in toxic drug users. Sequential retinal thickness analysis has revealed that rapid retinal thinning, which may follow retinal thickening, can indicate subclinical toxicity in hydroxychloroquine users, often manifesting several years before conventional clinical signs appear [22,23].

### 3.10. Vitreoretinal Interface Diseases

Retinal thickness analysis is crucial in diagnosing and monitoring vitreoretinal interface diseases, such as epiretinal membrane (ERM) and vitreomacular traction (VMT).

OCT reveals characteristic findings, including retinal thickening, the distortion of retinal layers, and tractional forces on the macula [58,59,60]. These features are crucial for diagnosing the disease, assessing its severity, and planning surgical interventions such as vitrectomy. In ERM, OCT retinal thickness maps also identify the extent of membrane and indicate the severity of its traction by the degree of retinal thickening [59]. In VMT, it visualizes focal or broad vitreoretinal adhesions and their impact on the underlying retina [58].

Regular OCT monitoring is indispensable in managing these conditions, as progressive traction can lead to thickness changes and even complications like macular holes or retinal detachment, necessitating timely surgical treatment [60].

### 3.11. Retinal Trauma and Post-Surgical Monitoring

Retinal thickness analysis is invaluable in assessing structural changes following ocular trauma or retinal surgery. In cases of blunt or penetrating trauma, OCT helps detect subtle macular disruptions, such as commotio retinae, subretinal hemorrhage, macular holes, or retinal detachments [61]. Retinal thickening or thinning in specific layers, such as the outer retinal or photoreceptor layers, can provide insights into trauma severity and potential recovery [61,62].

Post-surgically, OCT is crucial in monitoring retinal reattachment, macular hole closure, and epiretinal membrane resolution. Additionally, retinal structural (remodeling) or photoreceptor recovery can be evaluated through retinal thickness or outer retinal thickness profiles, providing insights into visual prognosis [63]. In eyes with retinal detachment or macular edema, OCT identifies persistent subretinal or intraretinal fluid and tracks changes through thickness comparison, aiding in assessing improvement or worsening and guiding decisions for further intervention.

This application highlights OCT’s role in the acute and long-term management of traumatic and surgical retinal conditions.

### 3.12. Miscellaneous Retinal Diseases

Retinal thickness analysis is instrumental in diagnosing and managing various retinal conditions beyond the commonly discussed diseases. Notable examples include pseudophakic cystoid macular edema (Irvine–Gass syndrome) [64], central retinal artery occlusion (CRAO) [65], and anterior ischemic optic neuropathy (AION) [66].

Pseudophakic cystoid macular edema, a common cause of visual loss following cataract surgery, can be effectively diagnosed and monitored using OCT thickness measurements. In cases of central retinal artery occlusion, OCT enables the assessment of retinal thickness changes associated with CRAO, providing valuable information for diagnosis and monitoring. Similarly, in anterior ischemic optic neuropathy, OCT assists in evaluating retinal nerve fiber layer thickness, aiding in both diagnosis and tracking disease progression.

### 3.13. Retinal Manifestations of Systemic and Neurodegenerative Diseases

Retinal thickness analysis has emerged as a valuable tool in detecting and monitoring systemic and neurodegenerative diseases (Table 4) [67]. Alterations in specific retinal layers, notably thinning of the ganglion cell–inner plexiform layer (GC-IPL) and RNFL, have been associated with conditions such as Alzheimer’s disease, Parkinson’s disease, and multiple sclerosis [67,68,69,70,71].

In Alzheimer’s disease, studies have demonstrated significant correlations between retinal thinning and established biomarkers of neurodegeneration, including tau protein levels and hippocampal volume [67]. These findings suggest that retinal imaging can detect neurodegenerative changes even in cognitively asymptomatic individuals, highlighting its potential as a non-invasive biomarker for early diagnosis.

Similarly, in Parkinson’s disease, retinal imaging has revealed specific patterns of retinal thinning, which may reflect dopaminergic neuronal loss [68,69]. These retinal changes could serve as accessible biomarkers for disease progression and therapeutic efficacy.

In multiple sclerosis, retinal imaging has been utilized to assess retinal layer thickness, providing insights into neuroaxonal damage and correlating with disease activity and progression [70,71]. This underscores the utility of retinal imaging in monitoring neuroinflammatory conditions.

Retinal thickness analysis has indeed shown potential in monitoring thyroid-associated ophthalmopathy (TAO) [72]. Studies have reported macular thinning and variations in RNFL thickness in TAO patients, suggesting that these parameters could serve as adjuncts in evaluating disease activity.

The expanding application of retinal imaging in neurological and systemic diseases bridges the gap between ophthalmology and broader medical fields, facilitating interdisciplinary approaches to patient care. As research advances, retinal thickness analysis holds promise as a non-invasive, cost-effective biomarker for the early detection and monitoring of neurodegenerative diseases.

## 4. Challenges and Recent Advances

### 4.1. Challenges and Limitations

#### 4.1.1. Variability in Measurements

Retinal thickness measurements are subject to variability due to differences in OCT devices, patient-specific factors, and imaging conditions [73,74]. Variations in axial length, refractive errors, or inconsistent alignment during scans can lead to discrepancies in measurements [75,76]. Additionally, inter-device variability remains a challenge, with different OCT manufacturers employing unique algorithms for segmentation and thickness quantification [73]. This lack of standardization complicates comparisons across studies and limits the reproducibility of results.

#### 4.1.2. Artifacts and Segmentation Errors

Artifacts, including motion artifacts, poor signal strength, and media opacities, often compromise the quality of OCT images [77]. Segmentation errors, particularly in cases with severe pathology or poor image contrast, can lead to inaccurate measurements of retinal thickness [12,78]. Common issues such as the misidentification of retinal boundaries or incorrect layer assignments pose challenges for clinical and research applications, requiring manual correction that can be time-consuming and subjective [79].

#### 4.1.3. Challenges in Interpretation and Threshold Setting

Despite advancements, there is a lack of consensus on threshold values for defining disease severity or treatment response using retinal thickness analysis in many conditions. While CST is widely used to monitor macular edema, specific thresholds for intervention often vary among clinicians and clinical trials. For instance, ME was defined as a CST of 300 μm or more in some studies [32], whereas others used different thresholds [80], often depending on the OCT device used for measurement and its normative database. This lack of standardized interpretation criteria limits the generalizability of findings and complicates clinical decision-making.

### 4.2. Advances in Retinal Thickness Analysis

#### 4.2.1. Artificial Intelligence (AI)

The integration of artificial intelligence (AI) and machine learning (ML) into OCT analysis has transformed the diagnostic landscape for retinal diseases [81]. AI algorithms are increasingly employed for automated segmentation, reducing human error and improving consistency. Furthermore, ML models trained on retinal thickness maps or thickness analysis can classify diseases with high accuracy, predict disease progression, and assist in the early detection of conditions such as diabetic retinopathy or AMD. These advancements hold promise for personalized medicine by tailoring treatment plans based on AI-driven insights.

#### 4.2.2. Integration with Multimodal Imaging

Combining OCT thickness analysis with other imaging modalities, such as OCT angiography (OCTA), fundus autofluorescence (FAF), and fluorescein angiography (FA), provides a more comprehensive assessment of retinal conditions. For example, OCTA provides a non-invasive visualization of retinal vasculature, complementing thickness measurements in retinal vascular diseases such as retinal vein occlusion and diabetic retinopathy. This integration enables clinicians to correlate neuroretinal or thickness abnormalities with vascular changes, enhancing disease understanding and providing insights into diagnostic and monitoring strategies.

#### 4.2.3. Real-Time Monitoring and Telemedicine

Advances in portable and compact OCT devices have facilitated the real-time monitoring of retinal conditions in diverse settings, including rural and underserved areas. These portable systems, coupled with telemedicine platforms, enable remote interpretation by specialists, increasing accessibility to retinal care [82]. This is particularly valuable for patients with chronic conditions requiring frequent monitoring, such as DME and exudative AMD.

## 5. Future Directions

### 5.1. Standardization of Analysis Protocols

One of the key future directions in retinal thickness analysis is the development of standardized protocols for data acquisition, processing, and interpretation. Variability in measurements due to differences in OCT devices, segmentation algorithms, and imaging conditions remains a significant challenge. International guidelines that define consistent methods for measuring and reporting retinal thickness could improve comparability across studies and clinical practices. These efforts would also facilitate the integration of retinal thickness data into multi-center registries and clinical trials.

### 5.2. Personalized Treatment Strategies

The application of retinal thickness trends to guide personalized treatment is gaining attention in retinal disease management. The longitudinal analysis of retinal thickness changes offers valuable insights into individual disease progression and therapeutic responses. For instance, patients with DME or AMD may benefit from tailored anti-VEGF regimens based on their specific patterns of fluid resolution and retinal restoration. Furthermore, integrating genetic predispositions and systemic health factors with OCT thickness analysis has the potential to enhance the precision of personalized treatment strategies [83].

### 5.3. Broader Integration with Telemedicine Platforms

The integration of retinal thickness analysis with telemedicine platforms has the potential to revolutionize eye care delivery, particularly in remote and underserved areas. Portable OCT devices, combined with cloud-based image storage and real-time analysis, could facilitate remote monitoring by specialists. When incorporated into wearable devices or home-based OCT systems, these tools can capture dynamic changes in retinal thickness and other parameters. These systems are especially valuable for conditions requiring frequent follow-ups, such as DME and AMD, as they reduce patient burden while maintaining high standards of care and enabling timely, personalized interventions.

### 5.4. AI for Earlier Detection and Prognostication

AI holds immense potential for the early detection and prognostication of retinal diseases. AI algorithms trained on large datasets can identify subtle changes in retinal thickness that may precede clinical symptoms, allowing for earlier intervention. For instance, AI-driven systems could detect early DME or subclinical AMD, facilitating timely treatment. Additionally, predictive models could estimate disease progression and visual outcomes based on retinal thickness trends, supporting more informed clinical decision-making.

### 5.5. Integration with Multimodal and Functional Imaging

Future advancements may involve the seamless integration of retinal thickness analysis with other imaging modalities and functional tests, like multifocal electroretinography (mfERG). This multimodal approach would provide a comprehensive understanding of both structural and functional aspects of retinal diseases, enhancing treatment monitoring. For example, integrating retinal thickness trends with vascular density metrics from OCTA or localized electrophysiologic responses could open new horizons in understanding retinal vascular diseases.

### 5.6. Quantitative Biomarker Development

OCT-based biomarkers are increasingly used in clinical trials to assess treatment efficacy and predict outcomes. Metrics such as CST in ME and geographic atrophy area in AMD have become standard endpoints in clinical trials and research [84]. The development of robust, reproducible biomarkers for thickness parameters of specific layers (e.g., the outer nuclear layer or EZ) improves the precision of clinical research and facilitates the translation of findings into clinical practice.

## 6. Conclusions

Retinal thickness analysis has proven valuable in the diagnosis, monitoring, and management of retinal diseases, providing key insights into structural changes and disease progression. Its ability to deliver quantitative data has made it an essential tool in clinical practice, facilitating early detection, accurate disease characterization, and personalized treatment approaches. The integration of advanced technologies, such as artificial intelligence and telemedicine platforms, holds promise for further enhancing diagnostic and monitoring capabilities, ultimately improving patient outcomes.

As research continues to uncover new applications and refine existing methodologies, retinal thickness analysis is expected to be more widely utilized in other retinal and systemic diseases. Its expanding role in understanding systemic conditions, including neurodegenerative diseases, highlights its interdisciplinary potential. Moving forward, standardizing analysis protocols and incorporating personalized treatment strategies based on thickness trends could enhance its significance in modern ophthalmology.

## Figures and Tables

**Table 1:** Comparison of major OCT systems for retinal imaging.

OCT System	Technology Description	Typical Wavelength	Scan Speed (A-scans/s)	Axial Resolution	Advantages	Limitations	Clinical Applications	Example Systems
Time-domain OCT (TD-OCT)	Uses a moving reference mirror to measure time delays in light reflections.	~840 nm	~400–1000	~10–15 μm	Simple design	Lower scanning speed and resolution compared to newer systems.	Basic retinal structure assessment; initial disease screening.	Stratus OCT (Carl Zeiss Meditec, Oberkochen, Germany)
Spectral-domain OCT (SD-OCT)	Employs Fourier-domain detection to capture the interference spectrum without moving parts.	~840 nm	20,000–70,000	~5–7 μm	High resolution; faster acquisition enables detailed layer analysis	Limited penetration in highly pigmented tissues; field of view may be limited.	Detailed retinal layer analysis; early detection of retinal pathologies (e.g., macular degeneration, diabetic retinopathy).	Cirrus HD-OCT (Carl Zeiss Meditec, Oberkochen, Germany), Spectralis OCT (Heidelberg Engineering, Heidelberg, Germany)
Swept-source OCT (SS-OCT)	Utilizes a tunable (swept) laser that rapidly scans across a range of wavelengths.	~1050 nm	100,000+	~6–8 μm	Deeper tissue penetration; high-speed imaging; improved choroidal visualization	Higher cost; may have slightly lower axial resolution than SD-OCT in some systems.	Choroidal imaging; enhanced visualization in high myopia and other posterior segment diseases.	DRI OCT Triton (Topcon, Tokyo, Japan), PLEX Elite 9000 (Zeiss Meditec, Jena, Germany)
Ultra-widefield OCT	Modified scanning protocols (based on SD- or SS-OCT) to capture a larger field of view.	Varies (often 840–1050 nm)	Depends on the underlying system	Similar to base system (~5–8 μm)	Provides a broader retinal map, enabling imaging of peripheral retina	Potential trade-offs between field of view and resolution; fewer clinical validation studies.	Peripheral retinal pathology detection; comprehensive retinal mapping.	Optos OCT (Dunfermline, UK), Xephilio OCT-S1 (Canon, Tokyo, Japan)

**Table 2:** Parameters for retinal thickness analysis, definitions, and applications.

Parameter	Definition	Applications	References
Central subfield thickness (CST)	The average thickness of the central 1 mm area of the macula, measured from the internal limiting membrane (ILM) to the retinal pigment epithelium (RPE).	Used to assess and monitor macular diseases and evaluate treatment efficacy in diverse macular diseases.	[10,11,12,13]
Macular volume	The total volume of the macula, calculated within a specific area (e.g., the central 6 mm), encompassing all retinal layers.	Utilized in longitudinal studies to monitor disease progression in macular conditions.	[13,14]
Segmented layer thickness	Thickness measurements of specific retinal layers, such as the photoreceptor layer or the outer nuclear layer, derived from segmentation analysis.	Evaluated in degenerative conditions, such as retinitis pigmentosa and geographic atrophy, to study structural changes.	[15,16]

**Table 3:** Uses of retinal thickness analysis for the diagnosis and monitoring of retinal diseases.

Disease/Condition	Key Thickness Parameters	Diagnostic Applications	Monitoring Applications
Age-related macular degeneration	CST, intra- or subretinal fluid thickness or volume, drusen thickness or volume	Identifies early age-related (e.g., drusen) or atrophic changes.	Tracks response to anti-VEGF therapy and progression of atrophy.
Diabetic retinopathy (diabetic macular edema)	CST, macular volume	Detects clinically significant macular edema (CSME) and even subclinical retinal thickening.	Evaluates fluid increase/reduction and therapeutic efficacy.
Retinal vein occlusion	CST	Detects macular edema and hemorrhages.	Monitors treatment response and recurrence.
Inherited retinal diseases	Outer retinal thickness, thickness of a specific (outer) retinal layer	Diagnoses structural abnormalities in progressive diseases.	Tracks degenerative changes and evaluates therapy efficacy.
Uveitic macular edema	CST	Identifies inflammation-induced edema.	Assesses response to treatments.
Post-surgical outcomes	CST, fluid thickness	Evaluates recovery after procedures like vitrectomy.	Tracks resolution of macular edema or reattachment success.
Central serous chorioretinopathy	Subretinal fluid thickness, CST	Detects subretinal fluid accumulation.	Tracks resolution of fluid and recurrence.
Myopic maculopathy	CST, regional retinal thickness	Identifies vision-threatening complications such as myopic traction maculopathy and progressive macular atrophy.	Tracks progression of structural changes and complications, enabling timely surgical or pharmacological interventions.
Pediatric retinal diseases	CST, outer retinal layer thickness	Provides information on overall retinal and photoreceptor development/abnormalities. Identifies complications like neovascularization or retinal detachment.	Monitors retinal maturation and progression of inherited retinal diseases.
Retinal manifestations of systemic and neurodegenerative diseases	Ganglion cell–inner plexiform layer (GC-IPL) thickness, retinal nerve fiber layer (RNFL) thickness	Provides early non-invasive biomarkers of neurodegeneration such as Alzheimer’s and Parkinson’s diseases.	Tracks disease progression and neurodegenerative changes, facilitating interdisciplinary patient management.
Retinal drug toxicity	CST, parafoveal/perifoveal region thickness, EZ integrity/thickness	Detects early drug-induced outer retinal damage (e.g., plaquenil or pentosan polysulfate toxicity).	Monitors progression in overall retinal and photoreceptor damage. Guides timely interventions for subclinical toxicity.
Vitreoretinal interface diseases	CST, pattern/severity of retinal thickening on thickness map	Identifies structural changes such as focal adhesions or severe retinal distortion. Helpful for diagnosis of epiretinal membrane (ERM) and vitreomacular traction (VMT) by focal or diffuse macular thickening/elevation.	Tracks progression of tractional forces, retinal thickening, and complications (e.g., macular holes), assisting in planning surgical intervention when needed.

**Table 4:** Key retinal thickness parameters and their diagnostic and monitoring applications across various systemic and neurodegenerative diseases.

Disease/Condition	Key Thickness Parameters	Diagnostic Applications	Monitoring Applications
Thyroid Eye Disease	Variations in retinal thickness, including localized thickening or thinning, potentially due to inflammatory and compressive effects on the retina.	May serve as a non-invasive biomarker for assessing disease activity and aiding in early diagnosis.	Useful for monitoring treatment response and disease progression.
Diabetes Mellitus	Retinal thickening due to macular edema; retinal thinning resulting from ischemia and neurodegeneration in advanced stages.	Assists in the early detection of diabetic retinopathy and macular edema.	Facilitates evaluation of treatment efficacy and progression of retinal changes.
Hypertension	Subtle alterations in RNFL thickness and overall retinal thinning, reflecting vascular changes and microangiopathic damage.	Supports assessment of hypertensive retinopathy and provides insight into systemic vascular health.	Aids in monitoring the impact of antihypertensive therapy on retinal vasculature.
Multiple Sclerosis (MS)	Thinning of the GC-IPL and RNFL, indicative of neurodegenerative processes.	Provides a non-invasive marker for neurodegeneration associated with MS.	Enables tracking of disease activity and therapeutic response.
Alzheimer’s Disease	Reduction in retinal thickness, particularly in the RNFL and GC-IPL, correlating with cognitive decline.	May serve as an early indicator of neurodegenerative changes associated with Alzheimer’s.	Potentially useful for monitoring disease progression and response to interventions.
Parkinson’s Disease	Thinning of specific retinal layers, including the RNFL and inner nuclear layer, reflecting dopaminergic neuron loss.	Assists in early detection and understanding of disease mechanisms.	Useful for assessing disease progression and effectiveness of treatments.
Systemic Lupus Erythematosus	Changes in retinal vasculature, including vessel density and perfusion alterations, detectable via OCT angiography.	Provides insights into ocular manifestations of systemic autoimmune activity.	Aids in evaluating the efficacy of immunosuppressive therapies on retinal health.
